# Vaccination in the Light of the Royal British Commission

**Published:** 1899-04

**Authors:** M. R. Leverson

**Affiliations:** Fort Hamilton, L. I., N. Y.


### VACCINATION IN THE LIGHT OF THE ROYAL BRITISH COMMISSION.

M. R. Leverson, M. D., Fort Hamilton, L. I., N. Y.

(Continued from March No., page 130.)

Q. 10,245. Ricord stated that if it should be shown that vaccination introduced syphilis, vaccination must be abandoned, and he afterwards declared that the fact that vaccination induced syphilis was established. The first statement was in a lecture at Hotel Dieu, Paris, April, 1862, and the second was in an address to the Academy of Medicine of Paris, May 19th, 1863 (*Journal de Connaissances Medicales*, March 10th, 1865).

Q. 10,255 (Chairman, quoting Fournier's work, before cited). "We are wanting in circumstantial and sufficient information about the epidemic of vaccinal syphilis of which fifty-eight soldiers of the Fourth Regiment of Zouaves were victims in 1800 in Algiers, etc." Mr. Tebb: "As you have referred to that I may mention that I have here a list of the names, grade, etc., of the unfortunate soldiers, and that a copy of this document was sent to the Local Government Board at the time the question was asked, and the facts were officially denied."

Qs. 10,261-9. Discussion as to receiving communications to Mr. Tebb from Herr G. F. Kolb, who in his lifetime was considered one of, if not the, most eminent statisticians in Europe; Mr. Herbert Spencer, and Rektor Siljeström.

Q. 10,270. Letter from Herr G. F. Kolb, of Munich, Member Extraordinary of the Royal Statistical Commission of Bavaria: "From childhood I have been trained to look upon the cow-pox as an absolute and unqualified protective. I have from my earliest remembrance believed in it more strongly than in any clerical tenet or ecclesiastical dogma. Open and acknowledged failures did not shake my faith. I attributed them either to the carelessness of the operator or the badness of the lymph. In course of time the question of vaccine compulsion came before the Reichstag, when a medical friend supplied me with a mass of pro-vaccination statistics, in his opinion conclusive and unanswerable.

"This awoke the statistician within me. On inspection I found the figures were delusive, and a closer examination left no shadow of doubt in my mind that the so-called statistical array of proof was a complete failure. My investigations were continued, but with a similar result. For instance, in the kingdom of Bavaria, into which the cow-pox was introduced in 1807, and where for a long period no one except the newly-born escaped vaccination, there were in the epidemic of 1871 no less than 30,742 cases of small-pox, of whom 29,429 had been vaccinated, as is shown by the documents in the State Department. When with these stern proofs before us of the inability of vaccination to protect, we reflect upon the undeniable and fearful mischief which the operation so often inflicts upon its victims, the conclusion forces itself upon us that the State is not entitled either in justice or in reason to put in force an enactment so directly subversive of the great principle of personal right."

Qs. 10,271-2 cites Mr. Herbert Spencer as opposed to compulsory vaccination, and wishes to put in the names and titles of works by Herr Kolb and by Rektor Siljeström, so that the Commission may see what manner of men these anti-vaccinators are. One object is because one of the most eminent vaccinators, Sir John Simon, has described them by such terms as "ignorant," "dishonest," "quacks," and "idiotic."

Q. 10,275. Herr Kolb was Statistician Extraordinary to the Royal Statistical Commission of Bavaria. He was a member of the Bavarian Parliament and a member of the German Diet, and he held a number of other appointments.

Qs. 10,277-82. Rektor Siljeström is a Doctor of Philosophy, and in 1838 accompanied the French scientific expedition on the corvette "La Recherche." He had written a letter on the statistical aspect of the case, but the Chairman ruled it could not be admitted.*

Mr. Tebb then gives information relative to the defence or insurance fund, and hands in a number of forms of application for admission signed by candidates. Frequently under the head of "remarks" the candidate explains his objections to vaccination, which are chiefly that a child had been injured, permanently ruined, or killed by vaccination.

They began on January 14th, 1889, and continued to March 14th, 1890.

Q. 10,285 (Chairman). "Supposing a person gives that statement as a reason for joining, after that do you pay all their fines?" "We pay their fines and we pay their law expenses."

Qs. 10,286-7. Witness proposes to bring cases of injury and death before the Commission, similar to those mentioned by

---

* Why not, if the Commission was seeking the truth? Rektor Siljeström is a Swede, and not subject to British jurisdiction.—M. R. L.

Mr. Hutchinson in his evidence before the Select Committee of 1871, where he stated that every week he had cases brought before him at the hospital of alleged injury caused by vaccination.

Qs. 10,288-9 (By Mr. Bradlaugh). Rightly or wrongly a large number of parents have alleged to witness as the reason for not having their children vaccinated, the injury which they allege had been caused by the vaccination of their children—a census has been taken in about eighty towns, villages, and districts in England, with a return of 2,138 cases of injury and 546 deaths recorded up to the end of 1889.

Q. 10,293 (Dr. Bristowe). "Are not those papers largely filled up by agents of the Anti-Vaccination Society?" "I do not think that any of them are filled up by agents of the Anti-Vaccination Society; they are sent by parents direct to our Secretary in London, and the Secretary corresponds with the parents and receives their subscription. They do not come from anti-vaccination leagues, because other leagues have their own defence funds. I may mention that a single notice in a paper will in a day bring from twenty to thirty applications from parents who are desirous of protecting their children from the perils of vaccination."

Qs. 10,292-5-7. There are a little over 1,000 members of the Defence Fund League; they have no canvassers. The way is generally this: poor parents are summoned and do not know how to pay a fine; they have heard of cases of injury, and, making inquiries, hear of the League and make application. Some of the letters are most touching, detailing their previous experience of vaccination. Mr. Young, the late Secretary of the Society, was of opinion he could get 10,000 members if the Society had funds; but for every 5s. the members pay, it costs the Society about 50 per cent. more.

Q. 10,299. Testimonies of parents who were prosecuted. Amongst the reasons assigned for the non-vaccination of their children is that of previous injuries in their families. Has newspaper clippings of thirty-six cases since January, 1889; but it is not likely that he gets a quarter even of those which are published.

Q. 10,302 (The Chairman). "But if there is an injury or illness of any kind following the vaccination, the very literature you have spread broadcast would be very apt to make people think that vaccination was the cause of the injury, would it not?" "Yes; no doubt to some extent."

Q. 10,305. Then the census returns. It is far more voluminous; they are statements which have been communicated by householders in different parts of the country.

Q. 10,306 (Dr. Bristowe). "And consequently of no particular authority?" "Yes, there is a sort of authority as you will see."

Q. 10,307. "All of the committees consisting of anti-vaccinators?" "Yes, all the committees being pronounced anti-vaccinators who were willing to give their time and services in order to get this law repealed."

Q. 10,309. These are the sources of his reply to Mr. Bradlaugh (10,289) of 2,138 cases of alleged injury and 546 deaths reported up to the end of last year. Mr. Tebb has not examined them; he brings them just as they came in, that the members of the Commission may see for themselves exactly what they are.

Qs. 10,311-2. An examination into the papers is made in each town, the facts verified, and generally published in the local journals and a scrutiny solicited. These censuses have now been carried on for some years, yet in only one instance has their truthfulness been challenged. In 1886 Mr. Tebb wrote to *The* (London) *Times*, furnishing the results up to that date. Dr. Saunders in reply challenged the facts. The witness produced a copy of Dr. Saunders' letter, and of his reply, in which he established the accuracy of the returns to the satisfaction, he thought, of any reasonable person.

Qs. 10,313-6. Introduced the proceedings at public meetings of the Society for the Abolition of Compulsory Vaccination, for the purpose of showing the enormous amount of public opinion against it. Thousands of petitions have been presented to Parliament to repeal the law, and not one in favor of it by any persons unconnected with the medical profession. The next point he proposes to give is the testimony of the late Mr. Thomas Baker, barrister-at-law, and of the late Wm. Young, Scretary of the Society, written on his dying bed.

Q. 10,317. Evidence of Wm. Young, pharmaceutical chemist, No. 77 Atlantic Road, Brixton, September 12th, 1889: "I am the Secretary of the London Society for the Abolition of Complsory Vaccination, prior to which I was Honorary Secretary of the Anti-Compulsory Vaccination and Mutual Protection Society, etc. (naming three societies). For more than twenty years I have paid great attention to the subject of vaccination and its compulsory enforcement. Many cases of death directly resulting from vaccination have come under my personal observation, and where practicable I have in such cases endeavored to get official inquiries or coroner's inquests held, but not always with success. The late coroner, Dr. Lankester, would never, if he could help it, hold an inquest on the body of a child alleged to have been killed by vaccination. I have seen hundreds of cases of skin diseases, abscesses, erysipelas, pyæmia, bad eyes, etc., caused by vaccination. I have made it a practice to ask mothers why they had their children vaccinated. Their almost invariable replies have been, 'We hate it, and would not have it done unless compelled by law.' Others have told me how by various devices, such as (1) non-registration, (2) removal, (3) giving false addresses, (4) forging of certificate, they had escaped the law." That is all Mr. Young was able to write.

Next Mr. Tebb handed in an official report of Dr. Airey on three cases of fatal erysipelas following re-vaccination in the Sudbury Union; next an extract from *The* (London) *Lancet* of November 2d, 1889, of an infant dying of trismus which had supervened upon inflammation of the arm, the result of vaccination. The same case was reported in *The Lancet* of November 9th. From the date when the child's arm "took" it began to ail, and when first seen with trismus about three weeks after the performance of the operation, the vaccination marks had sloughed, coalescing and leaving a somewhat considerable cavity. *The Lancet* then goes on to argue that as six children were vaccinated with the same lymph without injury, we are bound to assume that the lymph was not the cause of the mischief! [And this from a medical journal conducted by a physician, who then assumes that the child had been either "recently exposed to the influence of some other morbid or specific infection, or is actually incubating such infection."*—Ed.]

Mr. Tebb continues: A correspondent informed him that the German disasters already before the Commission are unimportant compared with many others brought before the German Commission. On applying to the President of the Berlin Board of Health for further information he was informed that they could not furnish it to private individuals but that the Commission can obtain same through a diplomatic arrangement set of foot for that purpose. He also presents an analysis of a petition to the Reichstag, in 1881, for the repeal of the vaccination law.

---

* This pseudo-science has been improved upon by American physicians. In one case it was learnedly declared that the child (dead from vaccination), was suffering from latent scarlatina. In another, a case of trismus, which occurred in Brooklyn, N. Y., that the germs of tetanus must have got into the wound by exposure, the soil of Long Island being known to be infected with such germs. Then why inflict the wound in Long Island? And yet surely all these pseudo-scientists know that a quantity of a poison, to wit, Opium, which may not injuriously affect one person may kill another; but Opium is a poison none the less.—M. R. L.

